# A quality control portal for sequencing data deposited at the European genome–phenome archive

**DOI:** 10.1093/bib/bbac136

**Published:** 2022-04-18

**Authors:** Dietmar Fernández-Orth, Manuel Rueda, Babita Singh, Mauricio Moldes, Aina Jene, Marta Ferri, Claudia Vasallo, Lauren A Fromont, Arcadi Navarro, Jordi Rambla

**Affiliations:** European Genome-phenome Archive (EGA) in the Centre for Genomic Regulation (CRG), the Barcelona Institute of Science and Technology Dr. Aiguader 88, Barcelona, 08003 Spain

**Keywords:** Fastq, quality control, variant call format (VCF), binary alignment map (BAM), European Genome-Phenome Archive (EGA)

## Abstract

Since its launch in 2008, the European Genome–Phenome Archive (EGA) has been leading the archiving and distribution of human identifiable genomic data. In this regard, one of the community concerns is the potential usability of the stored data, as of now, data submitters are not mandated to perform any quality control (QC) before uploading their data and associated metadata information. Here, we present a new File QC Portal developed at EGA, along with QC reports performed and created for 1 694 442 files [Fastq, sequence alignment map (SAM)/binary alignment map (BAM)/CRAM and variant call format (VCF)] submitted at EGA. QC reports allow anonymous EGA users to view summary-level information regarding the files within a specific dataset, such as quality of reads, alignment quality, number and type of variants and other features. Researchers benefit from being able to assess the quality of data prior to the data access decision and thereby, increasing the reusability of data (https://ega-archive.org/blog/data-upcycling-powered-by-ega/).

## Introduction

Next-generation sequencing (NGS) has become the leading method for deoxyribonucleic Acid (DNA) sequencing due to its capacity to process millions of DNA molecules in a single experiment. Despite its wide use, NGS still suffers from several concerns. At the experimental (wet-lab) level, NGS has been shown to display random errors and systematic biases, including: polymerase chain reaction amplification problems, GC-content (or guanine-cytosine content) shift and contamination [1]. Some of these aspects can be quality controlled *a posteriori* using bioinformatics tools [2]. Historically, quality control (QC) has been based on checking ‘reference’ values for chosen parameters, yet recently algorithms based in machine learning methods have been introduced [3, 4]. Not only the experimental part of NGS is prone to errors, the downstream analysis of the raw data (dry lab) in the form of ‘pipelines’ (including genome mapping, variant calling, etc.) can create biases as well. Therefore, a robust bioinformatics-based QC is a critical step to the correct interpretation of sequencing results.

The European Genome–Phenome Archive (EGA) is a public repository of human genomic and phenotypic data, tasked with the secure maintaining and distributing of data. EGA is maintained in collaboration between the European Bioinformatics Institute and the Centre for Genomic Regulation [5, 6]. Since its launch in 2008, the EGA has been leading the archiving and distribution of human identifiable genomics data that require controlled access. To date, over 4890 studies, containing up to 7690 datasets, have been deposited and are available to authorized researchers.

In an effort to facilitate researchers the selection of the fittest data for their analysis, the EGA has assembled a set of QC procedures for the file formats where data are stored and distributed (i.e Fastq, Sequence Alignment Map (SAM), Binary Alignment Map (BAM), Compressed version of BAM (CRAM) and Variant Call Format (VCF) files). Reports resulting from these procedures are embedded in HTML and available through the main EGA website. EGA users can visualize the main attributes of the deposited files and obtain an overall idea about its quality and potential reusability before starting the access requesting process.

## Methods

### Stored files at EGA

One particular challenge faced by the EGA is how to deal with the great diversity of file formats submitted to it. For nonphenotypic data, the EGA accepts a variety of formats^1^ that span all the way from raw data (microarray, Illumina Fastq and Complete Genomics) to processed ones like BAM, CRAM or VCF. In that sense, EGA is a driver project for the Global Alliance for Genomics and Health and verifies the deposited data following the specifications for BAM/VCF data.

Performing QC for these different formats requires the use of different tools. We focused our analyses on the file formats that are popular in bioinformatics and that have been deposited at the EGA in significant numbers^2^. At the time of planning for the file QC procedures, Fastq, BAM/CRAM and VCF account for more than 60% of all files deposited at EGA.

#### File formats analysed and selected tools

Fastq is the *de facto* standard format for storing the output of NGS instruments. It combines the sequence as well as an associated per base quality score (PHRED score) of a base call. For Fastq files, QC parameters commonly checked include (i) filtering low-quality reads (according to thresholds in PHRED scores) in order to avoid subsequent false positives, and (ii) keeping contamination and other features under control [2].

Several options exist to perform QC on Fastq files [2, 7, 8]. The EGA has selected *FastQC*^3^ because it encompasses most of the desired QC options (e.g. quality score/base-call distribution, detection of contaminants such as adaptors and detection of duplicates, etc.) and it is recognized as the gold standard tool by the community. Reports generated by FastQC include a section with basic statistics like a number of sequences, % GC, etc., followed by some plots showing diverse features.

SAM is a format for storing read alignments against reference sequences. BAM and CRAM format files are compressed versions of SAM [9, 10]. Popular QC parameters checked include coverage, length of reads and percent of mapped reads. Among stats for QC in SAM/BAM/CRAM, those usually checked are: nonreference allele frequency, depth distribution, stats by quality and per-sample counts and singleton abundance. Taking into account the diverse options available to perform QC on aligned files [11, 12], the EGA has chosen *SAM tools* as a QC tool for alignment formats, as it allows extracting all these features in an efficient way while being a gold standard in bioinformatics analysis [13].

VCF format is a format for storing genomic variations such as single nucleotide polymorphisms (SNPs), insertions, deletions and structural variants together with annotations [14]. Interesting tags for QC that may be taken into account for VCF files are allele frequency, depth distribution, stats by quality and per-sample counts and singleton stats [11, 15]. The tools selected by EGA are *vcf tools* (options: —TsTv-summary, —SNPdensity 1000, —site-quality, −freq) and *bcf tools* (options: stats).

### File QC implementation

The EGA File QC portal has two components: (i) the QC pipeline, that uses the set of tools described above to generate a report for each file. (ii) The frontend pages, which presents the report results in user-friendly graphics, also per each file. The front end generates an HTML5 webpage using the Django Python framework, and the D3 Javascript library. The front-end webpage is inspired by the existing http://iobio.io/ website for BAM and VCF files adding some custom features [16].

## Results

QC reports can be accessed by anonymous EGA users from ega-archive website, browse-dataset-files page, where we present summary-level information about each dataset file and reference to its corresponding QC report. Researchers can also access each QC report, directly from a reference found in a paper or by browsing the EGA catalog.

**Figure 1:**
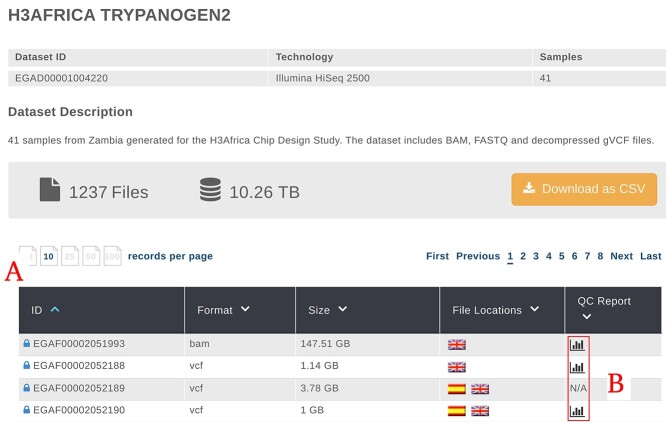
EGA website. Primary file information and how to access the QC report. (**A**) List with the EGA ID files composing the dataset. (**B**) Link to the File QC for each specific ID (https://ega-archive.org/datasets/EGAD00001004220/files).

As a typical user journey, a user could start querying for key tags in the search box on the main page of the EGA website (we searched for H3AFRICA). Among the results, there is a list of datasets matching the query (we selected H3AFRICA TRYPANOGEN2). Every dataset details page shows a ‘Browse Files’ button that forwards the user to the list of files available for that dataset (Figure 1). Once there, a table provides information related to the files (Figure 1A) including a link to the QC report when available (Figure 1B). By clicking on the link, the File Portal pops up for the selected EGAF file (VCF example: https://filesportal.ega-archive.org/EGAF00002052188).

Also, in ega-archive-org navigation header, About Section, we have added the ‘Quality Control Reports’ (https://ega-archive.org/about/quality-control-reports). Here, we detail on how to access and use QC reports, with a step-by-step guide.

### BAM/VCF QC report description

**Figure 2:**
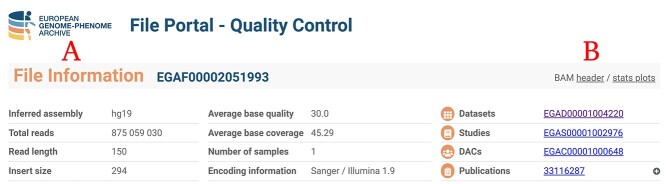
QC File Information section for a BAM file from H3AFRICA TRYPANOGEN2. (**A**) File Information section with general data about the bam file. (**B**) Link to bam header and plots generated by bamstats plot plugin from SAM tools (https://filesportal.ega-archive.org/EGAF00002051993).

For BAM and VCF files, the report is divided into two sections (Figure 2). ‘File Information’ (Figure 2A) shows some sample attributes, as well as information about the origin of the dataset and study. Furthermore, information about publications in which that file has been included is enclosed. The header of the BAM/VCF files is also viewable (Figure 2B), allowing the user to get an idea about which workflow and tools were used to generate that specific file. For BAMs, the link to stats plot displays the results of the plot bamstats script according to the SAM tools reference manual (Figure 2B).

**Figure 3:**
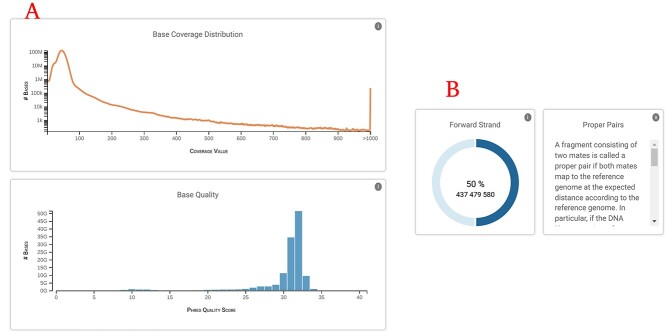
Left. Detailed QC plots for BAM files. (**A**) Base coverage distribution and base quality plots. (**B**) Example description for forward strand plot and pie chart showing % of proper pairs found in the H3AFRICA TRYPANOGEN2 BAM file (https://filesportal.ega-archive.org/EGAF00002051993).

The second section shows different plots depending on the type of file (Figure 3). BAM files comprise a series of plots giving information about base coverage distribution/quality, number of mapped reads, singletons and duplicates, among others (Figure 3A). For VCF files, included plots are: variant types, Ts/Tv ratio and variants quality. Details about each plot are available by clicking the information icon included in each plot. Such explanations help to understand/interpret them (Figure 3B).

On the upper right side of each plot shown, there is an information tag which opens a detailed description of every plot. This may help users to understand it.

## Discussion

Currently, there are close to 4 million files, summing 14 PB of data stored at the EGA. Up to November 2021, nearly 70% of them came from NGS sequencing results.

**Figure 4:**
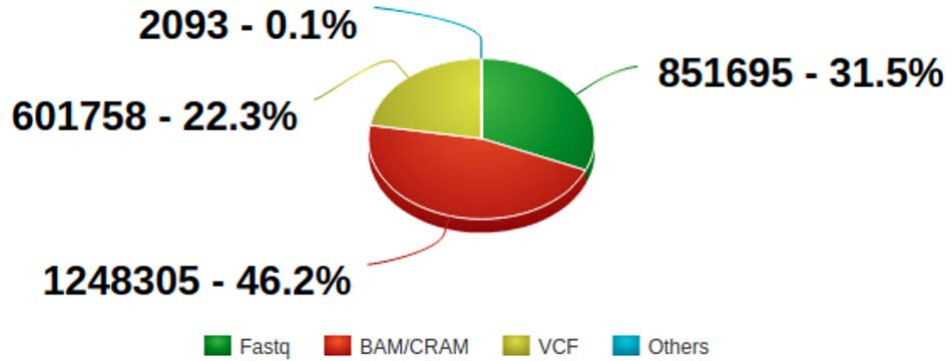
Pie chart showing number and percentages of NGS files at the EGA (update November 2021). Source: https://ega-archive.org/about/ega-statistics.

As shown in Figure 4, Fastq, BAM/CRAM and VCF constitute 99% of the total number of files, and approximately 91% of them have been analysed by the File QC portal workflow successfully. Those failing the QC are usually due to being corrupted or showing unexpected results. These files are then flagged and potential issues are resolved after requesting information from the submitters.

The File QC portal allows for checking the quality of the files, prior to downloading them. *On par* with the community, we run FastQC, SAM tools and BCF tools on deposited files, as these tools allow us to obtain robust statistics about the quality of the files. For each plot displayed, general feedback on the data is given in the information section, which enables the research community to instantly decide if the data are of suitable quality for their research purpose(s).

In order to decide if a file fulfills the quality criteria, it is recommended to check the ‘File Information’ section (Figure 2) as it allows detecting its main characteristics at a glance. Each plot is designed to check for different file parameters. A short text explaining how to interpret each plot is provided, helping the researcher to decide the suitability of the file (Figure 3B).

Files are not classified or tagged as having ‘good’ or ‘bad’ quality as that criteria depends on the purpose of the analysis to be done and should be applied by the potential requester.

We welcome users to contribute or suggest additional features to be evaluated and added to our File QC procedure and report. Suggestions can be made by contacting the EGA’s Helpdesk staff. Future implementations of QC on other file formats such as genome-wide association studies in *plink* format are under development. Regarding Fastq files, integration of MultiQC results for englobing all files within the same dataset is under study [17]. The File QC reports are free, publicly available and an open-source licensed resource.

**Key Points**

- We present a new File QC portal, ready to be used for any desired deposited dataset by the research community to check if Fastq, BAM/CRAM and VCF files within the EGA dataset fulfill applicants’ quality requirements to be used in their own analysis.
- We analysed quality parameters for more than 1 500 000 files stored at the EGA comprising Fastq, BAM/CRAM and VCF files.
- We used FastQC, SAM tools and BCF tools/VCF tools as gold standard open source tools for checking the quality and generated user-friendly plots to allow users an easy interpretation.

## Footnotes

1
https://ega-archive.org/submission/sequence

2
https://ega-archive.org/about/ega-statistics

3
http://www.bioinformatics.babraham.ac.uk/projects/fastqc/

## Notes

**Dietmar Fernández-Orth** is a postdoctoral bioinformatics researcher at the European Genome-phenome Archive (EGA) based at the Centre for Genomic Regulation (CRG) in Barcelona, Spain. His current research interest includes both technical and biological quality control of NGS deposited at the EGA.

**Manuel Rueda** is a senior computational biologist at the EGA. His current research is focused on the creation and dissemination of bioinformatics tools to enable community sharing of genomic data.

**Babita Singh** is a postdoctoral bioinformatics researcher at the EGA. Her current research is focused on developing tools and principles for ethical human genomics and phenotypic data sharing. She is involved in several EU H2020-funded projects related to genomics and diseases as well as a contributor to ELIXIR and Global Alliance for Genomics and Health (GA4GH) driver projects.

**Mauricio Moldes** is a technician supporting the EGA at the CRG, Barcelona.

Aina Jené is a bioinformatician currently working as a submission expert at the EGA based at the CRG, Barcelona.

**Marta Ferri** is a bioinformatician at the EGA at the Centre for Genomic Regulation (CRG), Barcelona. Her current work is based on the analysis and processing of genomic and phenotypic data.

**Claudia Vasallo** is a Postdoctoral Bioinformatics Researcher at the EGA. Her current research is focused on the handling of genomic and phenotypic data and metadata in the EGA and in several EU H2020 projects where EGA participates. She also contributes to ELIXIR and GA4GH initiatives.

**Lauren A. Fromont** is a scientific project manager at the EGA. She is coordinating projects related to data access and discovery, from internal projects to EU H2020-funded projects in health and genomics. She also contributes to ELIXIR and GA4GH projects.

**Arcadi Navarro** is an ICREA research professor at Pompeu Fabra University (UPF) and Group Leader of the EGA team at the CRG. Since 2019, he is also the director of the Barcelonaβeta Brain Research Center, Pasqual Maragall Foundation. His research interests include genome evolution, computational genomics, chromosomal speciation, genotypes and phenotypes, and the biological roots of senescence.

**Jordi Rambla** is the team leader of the EGA group at the CRG. In addition to managing both the research and the development sides of the EGA, he is a collaborator on several ELIXIR initiatives, leading the GA4GH Beacon API team and ELIXIR Beacon, and EGA/EVA/ENA driver project champion. He also contributes to several GA4GH initiatives like Discovery WS, Crypt4GH, htsget and rnaget.
